# Isolation and characterization of microsatellite markers for the threatened African endemic tree species *Pterocarpus erinaceus* Poir.

**DOI:** 10.1002/ece3.6944

**Published:** 2020-10-27

**Authors:** Benziwa Nathalie Johnson, Marie Luce Akossiwoa Quashie, Gilles Chaix, Letizia Camus‐Kulandaivelu, Kossi Adjonou, Kossi Novinyo Segla, Adzo Dzifa Kokutse, Christine Ouinsavi, Babou André Bationo, Habou Rabiou, Kouami Kokou, Hélène Vignes

**Affiliations:** ^1^ Laboratoire de Recherche Forestière (LRF) Université de Lomé Lomé Togo; ^2^ CIRAD, UMR AGAP Montpellier France; ^3^ AGAP Univ Montpellier CIRAD, INRAe Institut Agro Montpellier France; ^4^ Laboratoire d'Etudes et de Recherches Forestières Faculté d'Agronomie Université de Parakou Parakou Bénin; ^5^ Institut de l'environnement et de recherches agricoles (INERA) Burkina Faso; ^6^ Faculté des Sciences Agronomiques (FSA) Université de Diffa Diffa Niger

**Keywords:** African tree species, genetic diversity, next‐generation sequencing (NGS), nuclear microsatellites, *Pterocarpus erinaceus*

## Abstract

To study the genetic diversity and structure of the forest species *Pterocarpus erinaceus* Poir., seventeen polymorphic nuclear microsatellite markers were isolated and characterized, using next‐generation sequencing. Three hundred and sixty‐five (365) individuals were analyzed within fifteen (15) West African populations. The number of alleles for these loci varied from 4 to 30, and the heterozygosity varied from 0.23 to 0.82. The seventeen (17) primers designed here will allow characterizing the genetic diversity of this threaten species on its natural stands and to better understand the population differentiation mechanisms shaping it.

## Graphical abstract

In order to understand the genetic structure of populations and to assess the genetic diversity of Pterocarpus erinaceus (Fabaceae) in West Africa, genetic markers, in this case nuclear microsatellites, were isolated from individuals come from 4 countries, Togo, Benin, Burkina Faso, and Niger. The knowledge acquired will allow us to understand the functioning and evolution of the species in order to implement effective conservation strategies essential for the sustainable management of genetic resources.

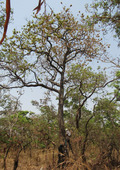

## 1 INTRODUCTION

*Pterocarpus erinaceus* Poir. (Lamarck & Poiret, 1823) is commonly known as African rosewood. This important tree belonging to the Fabaceae family is native from the Guinean forest–savannah mosaic ecoregion and has been reported from Senegal to Cameroon (Adjonou et al., 2019; Arbonnier, 2004; Giffard, 1974). Stands of this Leguminous species are especially targeted for timber and wood fuelwood production, but this species has also several medicinal uses (Fontodji et al., 2011; Kokou et al., 2009; Segla et al., 2015). Recent scientific investigations on *P. erinaceus* international trade have highlighted a considerable increase in export volume of its wood from West Africa countries for Asia, particularly China (Dumenu, 2019; Lawson, 2015). As a consequence of its overexploitation, the Convention on International Trade in Endangered Species of Wild Fauna and Flora classified the species as threatened (CITES, 2016) and it has become the focus of conservation concern in African countries. Because of its high‐quality wood, but also as a drought and fire‐resistant plant species with traditional medicine uses in sub Saharan Africa (Duvall, 2008; Karou et al., 2003; Ouedraogo et al., 2012), *P. erinaceus* is a good model species for the study of genetic diversity in *Pterocarpus* genus.

Understanding the dynamics of *P. erinaceus* populations’ evolution in West Africa in order to establish appropriate and efficient production and conservation strategies requires the study of its genetic diversity and structure on its natural stands. Among various molecular tools used to assess plant genetic diversity, microsatellite simple sequence repeats (SSR) markers are the most widely employed because they are codominant and possess high levels of polymorphism and stability (De et al., 2017; Morgante & Olivieri, 1993).

So far, only few studies have addressed the question of genetic diversity and population structure with the *Pterocarpu*s pantropical genus. Muller et al. (2006) have developed a set of eight (8) microsatellite markers for *Pterocarpus officinalis* Jacq., an important tree species of the Caribbean wetland forest. More recently, in order to facilitate population identification and biodiversity protection, Hong et al. (2020) have sequenced and analyzed the whole chloroplast genomes of five *Pterocarpus* species: *P. macrocarpus, P. santalinus, P. indicus, P. pedatus, and P. marsupium*. This study has also led to the description of chloroplastic SSR. The present study describes a new SSR set designed for *P. erinaceus* and its use to describe the genetic diversity of three hundred and sixty‐five (365) individual trees originating from West Africa. Owing to the laborious and expensive microsatellites development by conventional methods (Oliveira et al., 2006; Pimentel et al., 2018), we chose to use next‐generation sequencing (NGS) technologies. The major advantage of this approach is the identification of a large number of SSR allowed by the production an important volume of sequence data (Rico et al., 2013; Senan et al., 2014; Vieira et al., 2016).

## 2 MATERIAL AND METHODS

**Figure 1:**
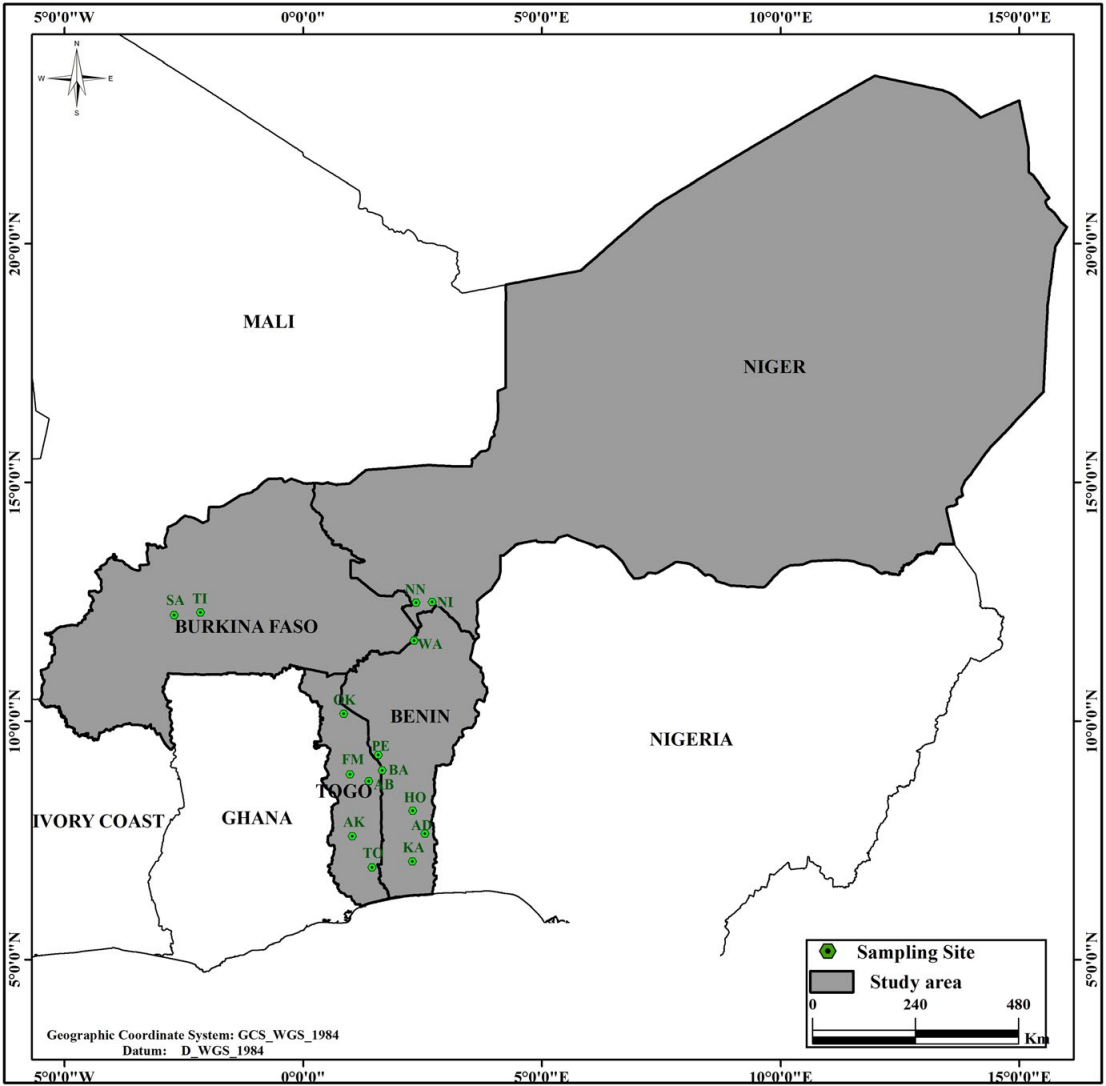
Map of study area and location of sampling sites in the four West Africa countries. Burkina Faso: SA, Sarya; TI, Tiogo. Togo: OK, Oti‐Kéran; FM, Fazao‐Malfakassa; AB, Abdoulaye; AK, Plateau Akposso; TO, Togodo. Benin: KA, Koussoukpa; AD, Adakplamè; HO, Houin; BA, Bassila; PE, Pénessoulou; WA, Bénin Parc W. Niger: NI, Niger Parc W; NN, Tamou

We sampled nine to thirty adult trees in 15 populations (Table 1) with a total of 365 trees in four countries of West Africa which are Benin, Burkina Faso, Niger, and Togo (Figure 1).

Freshly collected leaves were dried in a coffee filter containing 10 g Silica gel. Each filter containing sample was put in a plastic zip bag for transport to the laboratory. Our genomic library was constructed using DNA of *P. erinaceus* samples from twelve randomly selected individuals among populations (Table 1).

**Table 1:** Characteristics of sampled sites for *Pterocarpus erinaceus*: country location, name of sampling sites, number of trees sampled per site, and GPS coordinates

Country	Sampling sites	Number of trees sampled	GPS	
Niger	Parc W (NI) **	27	12°14′31.8″ N	2°17′33.4″ E
	Tamou (NN) **	14	12°35′55.1″ N	2°20′34.3″ E
Bénin	Koussoukpa (KA) *	25	7°03′27.8″ N	2°17′02.1″
	Bassila (BA) *	24	8°57′58.8″ N	1°39′01.9″ E
	Pénessoulou (PE) **	27	9°18′01.7″ N	1°23′40.1″ E
	Parc W (WA)	24	7°38′33.8″ N	2°19′22.0″ E
	Adakplamè (AD) **	27	7°38′33.8″ N	2°33′02.3″ E
	Houin (HO) *	24	8°07′34.5″ N	2°17′39.0″ E
Togo	Oti‐Kéran (OK) **	25	10°06′12.2″ N	0°41′32.5″ E
	Fazao‐Malfakassa (FM) *	30	8°30′31.7″	0°51′56.3″ E
	Abdoulaye (AB) **	30	8°40′30.2″ N	1°21′02.2″ E
	Plateau Akposso (AK)	23	7°35′27.6″ N	1°01′29.9″ E
	Togodo (TO) *	30	6°51′38.6″ N	1°30′45.4″ E
Burkina Faso	Sarya (SA) **	9	12°15′57″ N	2°08′47″ E
	Tiogo (TI) **	25	12°17′22″ N	2°47′12″ E
Total		365		

* and ** geographic origin of individuals selected for genomic library construction.

** geographic origin of individuals selected for the first screening.

### 2.1 DNA extraction

Total genomic DNA extraction was performed with a solution of alkyltrimethylammonium bromide (MATAB) using twenty milligrams of dried leaves from each tree sample. Extraction protocol used derived from Bousquet et al. (1990) methodology.

The quality of the genomic extracted DNA was controlled on a 1% agarose gel, and quantification was done by Hoechst assay using fluoroskan (Fluoroskan™ Microplate Fluorometer).

### 2.2 Cross‐amplification test of *P. officinalis* microsatellites markers on *P. erinaceus*

Cross‐amplification tests using the SSR developed by Muller et al. (2006) on *P. officinalis* were performed using *P. erinaceus* individuals from different sampling sites except for Tamou reserve (Niger). Loci were amplified by PCR in a 96‐well plate using 10‐μL volume reaction containing 20 ng of DNA, 1X reaction buffer B (Mg^2+^ free; 0.8 M Tris–HCl, 0.2 M (NH_4_)_2_SO_4_, 0.2% w/v Tween‐20; Solis BioDyne), 0.08 μM of the M13‐labeled primer, 0.1 μM of each primer forward fluorescent (FAM, NED, PET, and VIC) and the primer reverse, 0.1 μM of M13 fluorescent primer, 2 mM of MgCl_2_, 200 μM dNTPs, 0.4X Q‐solution (Facilitates amplification of GC‐rich templates; Qiagen^®^), 0.04 mg/ml of BSA solution (Bovine Serum Albumine; Qiagen^®^), and 0.06 U/µL of *Taq* DNA polymerase. PCR running conditions were as follows: initial denaturation at 94°C for 4 min followed by 36 cycles each at 92°C in 30 s, 1 min at 52°C, 45 s at 72°C, and with a final extension step at 72°C for 5 min. Electropherograms were analyzed, and allele sizes were determined using GeneMapper^®^ software version 4.1 using GeneScan 600 LIZ as a size standard (Applied Biosystems). Four out of the eight primers failed to amplify the target loci (mPoCIRE01, mPoCIRE04, mPoCIRH02, and mPoCIRE09), and two showed little polymorphism (Table 2).

**Table 2:** Results of cross‐amplification with *P. officinalis* microsatellites markers on *P. erinaceus* populations from Togo

	Niger	Bénin						Togo					Burkina Faso	
	Parc W	Koussoukpa	Bassila	Pénessoulou	Parc W	Adakplamé	Houin	Oti‐Kéran	Fazao‐M	Abdoulaye	Plateau Akposso	Togodo	Saria	Tiogo
mPoCIRE01														
Amplification percentage	NA	NA	NA	NA	NA	NA	NA	NA	NA	NA	NA	NA	NA	NA
Number of alleles	0	0	0	0	0	0	0	0	0	0	0	0	0	0
mPoCIRE04														
Amplification percentage	NA	NA	NA	NA	NA	NA	NA	NA	NA	NA	NA	NA	NA	NA
Number of alleles	0	0	0	0	0	0	0	0	0	0	0	0	0	0
mPoCIRF08*														
Amplification percentage	96.3%	92.0%	95.8%	96.3%	91.7%	40.7%	87.5%	100.0%	55.2%	26.7%	82.6%	77.4%	100.0%	100.0%
Number of alleles	1	1	1	2	2	1	1	1	1	1	2	1	1	1
mPoCIRH02														
Amplification percentage	NA	NA	NA	NA	NA	NA	NA	NA	NA	NA	NA	NA	NA	NA
Number of alleles	0	0	0	0	0	0	0	0	0	0	0	0	0	0
mPoCIRE09														
Amplification percentage	NA	NA	NA	NA	NA	NA	NA	NA	NA	NA	NA	NA	NA	NA
Number of alleles	0	0	0	0	0	0	0	0	0	0	0	0	0	0
mPoCIRH08														
Amplification percentage	77.8%	88.0%	75.0%	63.0%	70.8%	29.6%	50.0%	100.0%	55.2%	30.0%	82.6%	74.2%	100.0%	92.0%
Number of alleles	9	8	5	10	10	5	9	7	10	5	9	10	4	8
mPoCIRH07*														
Amplification percentage	96.3%	88.0%	87.5%	85.2%	87.5%	55.6%	91.7%	100.0%	69.0%	30.0%	87.0%	77.4%	100.0%	100.0%
Number of alleles	3	2	2	3	3	2	2	3	4	3	3	4	1	3
mPoCIRF03														
Amplification percentage	85.2%	68.0%	91.7%	40.7%	66.7%	29.6%	62.5%	72.0%	37.9%	20.0%	65.2%	51.6%	66.7%	88.0%
Number of alleles	9	6	5	7	7	7	8	11	9	4	7	6	4	5

* loci with little polymorphism; NA = no amplification

These eight loci were considered noninformative and showed insufficient variability to be used for genetic studies of *P. erinaceus*.

### 2.3 Construction of the DNA library and validation

The Westburg NGS DNA Library PrepKit was used to prepare the DNA library with a pooled DNA extract from twelve samples. The library was built following the manufacturer instructions. Using this kit, enzymatic fragmentation allows for obtaining DNA fragment sizes from 200 to 600 bp (suggested by the manufacturer) depending on the reaction time and the amount of DNA input. DNA was fragmented in an Eppendorf Mastercycler^®^ nexus using 35 µl volume of pooled DNA (1µg) to which 5 µl of ER/A‐tailing buffer (10X) and 10 µl of ER/A‐tailing enzyme mix (5X) were added. The fragmentation program used in the thermocycler included a first step of precooling at 4°C for 5 min followed by the second step of fragmentation with three fragmentation times, 1 min at 4°C, 10 min at 32°C, and 30 min at 65°C.

The sample was then ligated with Illumina adapters. The recommended Illumina indexes in addition to the Westburg kit come from the following reference: TruSeq RNA Single Indexes Set A 12 Indexes (ref 20,020,492). Ligation reaction was performed in a PCR tube containing 45µl of fragmented DNA to which was added successively 20 µl of ligation buffer (5X), 10µl of DNA ligase, 10 µl of water, and 2.5µl of DNA illumina adapter. Incubation of the ligation reaction was performed at 20°C for 15 min.

Purification on magnetic beads (Agencourt AMPure XP beads ‐A63881‐, Beckman Coulter) was performed before and after PCR. The amplification conditions included initial denaturation at 98°C for 2 min, followed by 7 cycles of 98°C for 20 s, annealing at 60°C for 35 s, elongation at 72°C for 30 s, and a final 1 min elongation step at 72°C.

The quality of DNA library was controlled using an Agilent 4,200 TapeStation with a screen tape D5000, and the fragments sized between 100 and 600 pb mainly, with an average of 260 bp. The DNA library (fragments) was quantified using the Takara kit (638,324) on a qPCR machine (LightCycler^®^ 480 Real‐Time PCR System, Roche Life Science).

### 2.4 Sequencing

MiSeq system Illumina sequencer DNA was used to perform DNA sequencing on the genotyping platform at CIRAD‐Montpellier. A 500 cycles NANO V2 cartridge Illumina (2 x 250 pb) was used to sequence the library.

### 2.5 Design and choice of primers

A total of 800,000 reads were generated for *P. erinaceus* DNA library. Development of optimized and streamlined microsatellites was based on a bioinformatics Galaxy pipeline and with following tools: FASTQ Groomer tool, Filter FASTQ tool, and ABySS parallel assembler (Simpson et al., 2009). The MISA MIcroSAtellite identification tool (Thiel, 2003) and primer modeling software Primer3 (Whitehead Institute) were used for the identification and design of microsatellites primers in the generated draft assembly. A data matrix containing all the microsatellite primers was obtained as output.

Among the 38,715 single sequence repeats identified, primers were designed for 11,718 sequence repeats of which 3,530 were dinucleotide repeats, 2,970 trinucleotide repeats, 2,847 tetranucleotide repeats, 1,001 pentanucleotide repeats, 525 hexanucleotide repeats, and 844 contained complex SSR motifs.

Thirty microsatellites were identified and selected for initial screening on the basis of the type and size of the repeat motif, as well as the annealing temperature as previously described Muller et al. (2006). Therefore, dinucleotide and trinucleotide SSRs with a repeat motif of 15 to 30 bp were randomly selected from those generated. The selected primers amplified SSR motifs with a minimum of five repetitions. The annealing temperature varied from 54 to 56°C, including that used by Muller et al. (2006) for *P. officinalis* (54°C). This first test was performed on an ABI 3500XL sequencer (Life technologies) using genomic DNA extract from eight individuals selected from different countries (Table 1).

An M13 tailed primer (5′‐CACGACGTTGTAAAACGAC‐3′), allowing detection of fluorescence, was added to the forward primers. Each PCR amplification was performed in a 96‐well plate using 10‐μL volume reaction containing 20 ng of DNA, 1X reaction buffer B (Mg^2+^ free; 0.8 m Tris–HCl, 0.2 m (NH_4_)_2_SO_4_, 0.2% w/v Tween‐20; Solis BioDyne), 0.08 μm of the M13‐labeled primer, 0.1 μm of each primer forward fluorescent (FAM, NED, PET, and VIC) and the primer reverse, 0.1 μm of M13 fluorescent primer, 2 mm of MgCl_2_, 200 μm dNTPs, 0.4X Solution “S” (additive solution that facilitates amplification of difficult templates; Solis BioDyne), and 0.05 U/µL of *Taq* DNA polymerase. PCR running conditions were as follows: initial denaturation at 94°C for 4 min followed by 36 cycles each at 92°C in 30 s, 1 min at 52°C, 45 s at 72°C, and with a final extension step at 72°C for 5 min.

The analysis of electrophoregrams with GeneMapper^®^ software version 4.1 using GeneScan 600 LIZ as a size standard (Applied Biosystems) allowed determining allele sizes. Among the 30 primer pairs tested, 17 were selected. Indeed, we eliminated primers with profiles that were difficult to read on GeneMapper®, or with no or little polymorphism. The 17 selected primers are shown in Table 4 and were used for screening the remaining individuals in order to calculate genetic parameters.

Genetic parameters including alleles’ number per locus, number of private alleles, observed heterozygosity (Ho), and expected heterozygosity (He) were computed using GenAIEx software version 6.0 (Peakall & Smouse, 2012). Deviation from the Hardy–Weinberg equilibrium (HWE) was measured for each locus by chi‐squared tests and p‐value significance assessed in the context of multiple testing with a Bonferroni correction procedure (Rice, 1989). Significant linkage disequilibrium was rated among these loci by using GENETIX software version 4.05 (Belkhir et al., 1996). MICRO‐CHECKER software version 2.2.3 (Van Oosterhout et al., 2004) was used to check for the null alleles in microsatellite data.

## 3 RESULTS AND DISCUSSION

Contrary to Hong et al. (2020) results in chloroplast genomes of five *Pterocarpus* species, among the single sequence repeats identified there was no single‐nucleotide repeats motifs accounted. The observed proportion of dinucleotide, trinucleotide, and tetranucleotide were respectively 30.1%, 25.3%, and 24.3%, while the tetranucleotide proportion was 2.27%. 4.5% of nucleotides were hexanucleotide repeats, and 7.2% were complex SSR motifs.

A total of 237 alleles were identified for the 17 locus on the 365 characterized trees, with each locus having from 4 to 30 alleles (mean of 13.9 alleles per locus) (Table 4). Table 3 shows the total numbers of specific alleles for each population (private alleles) across all 17 loci. A total of 34 private alleles have been identified out of the 15 populations. Their distribution varied from 0 allele (BA, AB, SA populations) to 13 alleles (OK population). The majority of them (22/34 alleles) was characterized by an allelic frequency smaller than 5%. The 12 remaining private alleles have a frequency varying from 5% to 12%. Mean values for the expected heterozygosity (He) varied from 0.42 to 0.65 (0.57 ± 0.02) and from 0.41 to 0.65 (0.55 ± 0.02) for observed heterozygosity (Ho) for the population screened (Table 3). Evidence of significant linkage disequilibrium was found for 12 out of 136 possible SSR pairwise combinations after Bonferroni corrections. Significant departures from Hardy–Weinberg equilibrium for 14 out of 17 loci were recorded after Bonferroni corrections, and presence of null alleles was suggested for all loci excepted for mPeCIR_D2 and mPeCIR_T3.

**Table 3:** Private alleles and heterozygosity levels across populations

Country	Sampling sites	Total number of alleles	Number of private alleles	Percentage of private alleles	Observed heterozygosity (Ho)	Expected heterozygosity (He)
Niger	Parc W (NI)	100	2	2%	0.58 ± 0.22	0.61 ± 0.19
	Tamou (NN)	79	3	3.7%	0.56 ± 0.24	0.57 ± 0.18
Bénin	Koussoukpa (KA)	85	3	3.5%	0.52 ± 0.33	0.52 ± 0.29
	Bassila (BA)	77	0	0%	0.50 ± 0.28	0.48 ± 0.25
	Pénessoulou (PE)	123	7	5.6%	0.63 ± 0.23	0.65 ± 0.22
	Parc W (WA)	96	2	2.1%	0.56 ± 0.22	0.62 ± 0.21
	Adakplamè (AD)	83	1	1.2%	0.50 ± 0.29	0.52 ± 0.27
	Houin (HO)	86	2	2.3%	0.49 ± 0.29	0.54 ± 0.28
Togo	Oti‐Kéran (OK)	113	13	11.5%	0.55 ± 0.28	0.65 ± 0.26
	Fazao‐Malfakassa (FM)	116	6	5.1%	0.65 ± 0.23	0.64 ± 0.23
	Abdoulaye (AB)	43	0	0%	0.41 ± 0.38	0.42 ± 0.23
	Plateau Akposso (AK)	89	1	1.1%	0.56 ± 0.26	0.57 ± 0.23
	Togodo (TO)	91	3	3.2%	0.52 ± 0.30	0.53 ± 0.28
Burkina Faso	Sarya (SA)	78	0	0%	0.58 ± 0.26	0.62 ± 0.20
	Tiogo (TI)	75	3	4%	0.61 ± 0.20	0.60 ± 0.16

**Table 4:** Characteristics of 17 microsatellite primers designed for *Pterocarpus erinaceus* Poir

Primer name	Primer sequences (5'−3')	Repeat motif	Allele size	TA°C	Na	Ho	He	p‐value
mPeCIR_D1	F: TTTCTTCTACTTTCCTTTCCC	(CT)_15_	109–124	54.4	16	0.70	0.74	0.000***
	R: AAGCAGGCTCAAGAGAAGA							
mPeCIR_D2	F: AACATGCAAGCAAAGCA	(AG)_13_	107–123	54.6	12	0.74	0.67	0.000***
	R: AAGGTGGAGCTAAAGAAGGT							
mPeCIR_D4	F: TCGGTTTTGGTCTTTGTG	(TC)_14_	152–167	55.4	16	0.82	0.78	0.000***
	R: CAGACCGTTGGGAAGAA							
mPeCIR_D5	F: TGTCCCGTGAAGAAAGG	(GA)_10_	102–159	55.3	10	0.43	0.38	0.000***
	R: AAGCAGGCTCAAGAGAAGA							
mPeCIR_D7	F: CGTCAGCCTCCAATCTC	(GA)_14_	189–203	54.9	20	0.69	0.68	0.002***
	R: CGCTTGATTTGGTCCTC							
mPeCIR_D8	F: CTCATGGGCACAGAACAA	(TA)_11_	177–205	56.4	30	0.71	0.75	0.008***
	R: GATGGGCTTCACAGCAA							
mPeCIR_D9	F: TTTCCCGGTGTCAAGAA	(TC)_16_	188–208	55.8	20	0.71	0.68	0.002***
	R: GACACACGCACATACAGAGA							
mPeCIR_D10	F: TCACCAAAACATGCACAA	(TG)_14_	214–230	55.1	11	0.46	0.52	0.000***
	R: GCTCATGCTTAGCCCC							
mPeCIR_D11	F: GGGTTAGAGTTTGAATGGG	(AG)_17_	221–239	54.5	22	0.75	0.78	0.000***
	R: GCCTTCCTCAGCACTATTT							
mPeCIR_D12	F: AACCTGCCCATCCATTT	(TC)_16_	238–253	56.1	10	0.52	0.54	0.000***
	R: TACACTGGGTCGTTGGG							
mPeCIR_D14	F: CAGCACTGGCACCAAC	(AG)_13_	280–307	55.1	29	0.76	0.78	0.000***
	R: CACCACACCGCTTAATGT							
mPeCIR_T1	F: TCCATTGGGGTATCTATGTG	(ATC)_6_	115–121	55.7	4	0.23	0.34	0.000***
	R: CCTCAAGGGTGTTTTGTGT							
mPeCIR_T2	F: ATCACGGGCTCTTCCTC	(TCT)_8_	121–130	56.0	9	0.38	0.43	0.000***
	R: TCATTGTTTCTGCAAATCCT							
mPeCIR_T3	F: GGCCATTCTTCATGTGTTT	(CTT)_8_	99–146	55.9	9	0.39	0.39	0.114ns
	GGAGATGGGTGAGAGTGAA							
mPeCIR_T4	F: CAGGAGGGGTGGTGG	(GAA)_6_	146–152	56.3	4	0.31	0.31	0.308ns
	R: GCATCCTAGCCCGATTT							
mPeCIR_T5	F: AGACCCGAACTTGTCCC	(TTA)_11_	145–167	55.7	11	0.48	0.61	0.459ns
	R: TGCCAGTGTGTGATGGA							
mPeCIR_T15	F: CCCTCATCAAGAAGAACCA	(ACA)_7_	277–295	56.0	4	0.29	0.33	0.000***
	R: CTTGCATCACCACCCTC							

Note

He, expected heterozygosity under Hardy–Weinberg equilibrium; Ho, observed heterozygosity; Na, number of individuals; TA°C, annealing temperature. p‐values for the Hardy–Weinberg Equilibrium test, significance threshold adjusted using sequential Bonferroni correction.

* *p* ≤ .05, ***p ≤ .001, ns = not significant.

Two populations of Togo, Oti‐Kéran and Fazao‐Malfakassa, and the population of Pénélessou in Benin have a particularly high rate of private alleles scoring respectively to 11.5%, 5.6%, and 5.1%. On the other hand, the populations of Abdoulaye (Togo), Bassila (Bénin), and Sarya (Burkina Faso) have no private allele. Observed heterozygosity generally follows the trend of private alleles level with high values for Oti‐Kéran (0.55), Fazao‐Malfakassa (0.65), and Pénélessou (0.63) compared to Abdoulaye (0.41) and Bassila (0.50) (Table 3). From a conservation perspective and on the basis of genetic diversity level, Oti‐Kéran, Fazao‐Malfakassa, and Pénélessou are the most interesting populations sampled in this study. These three populations notably come from protected sites (reserves and parks) located in the Sudanian area. On the contrary, Abdoulaye's samples come from the community forest exploited by the surrounding local populations following the principles of integrated and participatory management. The type forest management undergone by the tree populations may not be however the only explanative factor of the observed diversity levels. Studied populations all belong to natural stands distributed according to the climatic gradient of the Sahelian, Sudanian, and Guinean zones and exhibit high morphological variation. Johnson et al. (2020) identified 3 morphotypes for *P. erinaceus* in Togo characterized by phenotypic specificity related to a climate gradient. On the basis of descriptors related to leaves, fruits, and seeds, a morphotype adapted to the dry Sudanian zone was described in Oti‐Kéran and Fazao‐Malfakassa sites, a morphotype adapted to the semiwet Sudano‐Guinean zone was described in Abdoulaye site, and a third morphotype adapted to the wet Guinean zone was described in Akposso and Togodo sites. From this point of view, the level of genetic diversity could also be related to ecotypes but further work is requested to investigate this hypothesis. Finally, we would advise to account both for genetic diversity level and for ecotype specificity in future conservation programs for *P. erinaceus*.

This set of 17 specific primers of *P. erinaceus* would serve to study the genetic diversity of this species in West Africa. While the SSR set developed by Muller et al. (2006) on *P. officinalis* does not show a proper amplification and/ or polymorphism level on *P. erinaceus*, the transferability of the SSR markers set presented in this study on other *Pterocarpus* species should be further investigated.

## 4 CONCLUSION

In this study, thirty (30) microsatellites primers were developed based on *P. erinaceus* populations from three different African countries by using NGS (Illumina MiSeq sequencing technology). Seventeen (17) of these nuclear markers showed a high level of polymorphism in fifteen (15) locations, thus providing the first set of microsatellite markers for *P. erinaceus*. These microsatellite markers will be useful for characterizing genetic diversity and analyzing genetic structure for *P. erinaceus* populations in order to contribute to the implementation of optimal management and conservation plans for this species.

## CONFLICTS OF INTEREST

The authors have no conflicts of interest to declare.

## AUTHOR CONTRIBUTION

**Benziwa Nathalie Johnson:** Conceptualization (equal); Data curation (equal); Formal analysis (equal); Funding acquisition (equal); Investigation (equal); Methodology (equal); Project administration (equal); Resources (equal); Visualization (equal); Writing‐original draft (lead); Writing‐review & editing (equal). **Marie Luce Akossiwoa Quashie:** Conceptualization (equal); Funding acquisition (equal); Investigation (supporting); Methodology (equal); Project administration (lead); Supervision (equal); Visualization (equal); Writing‐original draft (supporting); Writing‐review & editing (equal). **Gilles Chaix:** Conceptualization (equal); Formal analysis (equal); Funding acquisition (equal); Investigation (supporting); Methodology (equal); Project administration (equal); Resources (equal); Supervision (equal); Validation (equal); Visualization (equal); Writing‐review & editing (equal). **Letizia Camus‐Kulandaivelu:** Formal analysis (equal); Validation (equal); Visualization (equal); Writing‐review & editing (equal). **Kossi Adjonou:** Conceptualization (supporting); Funding acquisition (equal); Investigation (supporting); Visualization (supporting); Writing‐review & editing (equal). **Kossi Novinyo Segla:** Conceptualization (supporting); Investigation (equal); Visualization (supporting); Writing‐review & editing (equal). **Adzo Dzifa Kokutse:** Conceptualization (equal); Funding acquisition (equal); Visualization (equal); Writing‐review & editing (equal). **Christine Ouinsavi:** Resources (equal); Writing‐review & editing (supporting). **Babou André Bationo:** Resources (equal); Writing‐review & editing (supporting). **Habou Rabiou:** Resources (equal); Writing‐review & editing (supporting). **Kouami Kokou:** Conceptualization (equal); Funding acquisition (equal); Visualization (equal); Writing‐review & editing (equal). **Hélène Vignes:** Data curation (equal); Formal analysis (equal); Investigation (equal); Methodology (equal); Resources (equal); Visualization (equal); Writing‐review & editing (equal).

## Data Availability

The data have been deposited with links to BioProject accession number PRJNA604893 in the NCBI BioProject database (https://www.ncbi.nlm.nih.gov/bioproject/). 17 Primers designed and evaluated: end of text (Table 4).
